# Performance of Routine *Helicobacter pylori* Invasive Tests in Patients with Dyspepsia

**DOI:** 10.1155/2013/184806

**Published:** 2013-12-18

**Authors:** Hsi-Chang Lee, Ting-Chang Huang, Chin-Lin Lin, Kuan-Yang Chen, Chung-Kwe Wang, Deng-Chyang Wu

**Affiliations:** ^1^Liver Center, Department of Gastroenterology, Ren-Ai Branch, Taipei City Hospital, Taipei 106, Taiwan; ^2^Division of Gastroenterology, Department of Internal Medicine, Kaohsiung Medical University Hospital, Kaohsiung 807, Taiwan; ^3^Department of Internal Medicine, Kaohsiung Municipal Hsiao-Kang Hospital, Kaohsiung 812, Taiwan; ^4^Cancer Center, Kaohsiung Medical University Hospital, Kaohsiung 807, Taiwan; ^5^Department of Medicine, Faculty of Medicine, College of Medicine, Kaohsiung Medical University, Kaohsiung 807, Taiwan

## Abstract

*Background*. This study was designed to compare the accuracy of three different invasive methods for the detection of *Helicobacter pylori (H. pylori)* infection in patients with dyspepsia. These tests included culture, histology, and the rapid urease test (CLO test). *Methods*. *H. pylori* infection was diagnosed prospectively in 246 untreated dyspeptic patients who underwent upper gastrointestinal endoscopy. The gold standard for *H. pylori* infection was based on a positive culture or both a positive histological examination and a CLO test. *Results*. *H. pylori* was diagnosed in 33.3% of the patients. The sensitivity, specificity, positive predictive value, negative predictive value, and overall accuracy were as follows: histology from the antrum (95.12; 95.12; 90.7; 97.5; 95.12%); histology from the antrum and corpus (95.12; 95.12; 90.7; 97.5; 95.12%); histology from the corpus (76.83; 96.95; 92.65; 89.33; 90.24%); culture (91.46; 100; 100; 95.91; 97.15%); a CLO test from the antrum and corpus (85.59; 100; 100; 93.71; 95.52%); a CLO test from the antrum (64.63; 100; 100; 84.97; 88.21%); a CLO test from the corpus (69.51; 100; 100; 96.77; 89.83%), respectively. *Conclusions*. Antral biopsy histology and culture are the best methods for the diagnosis of *H. pylori* infection in our cohort of patients with dyspepsia.

## 1. Introduction

*Helicobacter pylori* infection is very common worldwide, occurring in 40% to 50% of the population in developed countries, in 80% to 90% of the population in developing regions [1], and about 50% of the population in Taiwan [2]. The infection causes chronic gastritis which significantly increases the risk of developing gastric or duodenal ulcer [3, 4], gastric adenocarcinoma, and mucosa-associated lymphoid tissue (MALT) lymphoma [5, 6]. As the eradication of *H. pylori* has been shown to improve the outcome of peptic ulcer disease in terms of recurrence and complications, the accurate diagnosis of *H. pylori* infection is of clinical importance.

Several methods have been developed for the detection of *H. pylori* infection. These methods include noninvasive tests that are based on the detection of antibodies to *H. pylori* or the urea breath test (UBT) or invasive tests that require endoscopy to obtain tissue biopsies, such as the rapid urease test (RUT), culture, and histological examination [7–9]. Each test has advantages and disadvantages, which make it more or less appropriate for different situations. Of all the available tests, invasive tests are considered the most accurate. However, invasive tests are mainly limited by their proneness to sampling error, because of the patchy distribution of the bacteria throughout the stomach [10, 11]. These circumstances yield the possibility of false negative results if the biopsy is taken from the antrum or the corpus alone. Studies on biopsy sites for the diagnosis of *H. pylori* infection are sometimes conflicting. Antrum biopsy is recommended by Genta and Graham [12], while others recommend at least one corpus biopsy [13, 14]. So far no optimal biopsy site for the diagnosis of *H. pylori* status has been currently established.

This study has been designed and undertaken to compare the sensitivity, specificity, and accuracy of different invasive tests and biopsy sites for the diagnosis of *H. pylori* infection in clinical practice.

## 2. Methods

Patients with dyspepsia undergoing upper gastrointestinal endoscopy at Taipei City Hospital Ren-Ai Branch, Taipei, Taiwan, between March 2013 and July 2013, were included in this study. According to the Rome III criteria, dyspepsia is defined as one or more of the following symptoms: postprandial fullness, early satiation, and epigastric pain or burning [15]. Exclusion criteria were the following: bismuth salts, proton pump inhibitors, or antibiotic therapy within the last 2 months, previous *H. pylori* eradication therapy, chronic use of corticosteroids or immunosuppressants, prior gastric surgery, the presence of a bleeding peptic ulcer, severe concomitant disease, and pregnancy or lactation. All patients were informed of the objective of the study and subsequently gave informed consent in writing. This study was approved by the Ethics Committee of the Taipei City Hospital.

During endoscopic examination, several biopsy specimens were taken from each patient for histological examination: two from the antrum, one from the incisura angularis, and one from the corpus. For the rapid urease test, one was taken from the antrum and one from the body. For culture, one specimen was taken from the antrum.

### 2.1. Diagnostic Methods

#### 2.1.1. Histology

Biopsy specimens were fixed in formalin and sections were assessed for the presence of *H. pylori* by a modified Giemsa stain. The degree of inflammatory cell infiltration, atrophy, and intestinal metaplasia was assessed in sections stained with hematoxylin and eosin (H&E). The histological features of the antrum and body of the gastric mucosa were graded according to the updated Sydney System.

Histology (antrum) included two biopsy specimens from the antrum and one from the incisura angularis (the lesser curvature). Histological examination of two biopsy specimens from the corpus was also carried out.

#### 2.1.2. Culture

One antrum biopsy specimen from each patient was cultured. The specimen was rubbed across the surface of a CampyBAP agar plate (Brucella agar (Difco) + IsoVitaleX (Gibco) + 10% whole sheep's blood), and the plate was incubated at 35°C under microaerobic conditions (5% O_2_, 10% CO_2_, and 85% N_2_) for 4-5 days. The culture was considered to be *H. pylori* positive if one or more colonies of spiral or curved Gram-negative, oxidase (+), catalase (+), and urease (+) rods were present.

#### 2.1.3. Rapid Urease Test

Two biopsy specimens, one from the antrum and one from the corpus, were tested from each patient. The specimens were subjected to a rapid urease test (CLO test, Kimberly-Clark, USA), to detect the presence of *H. pylori* urease. A positive result was reported if the color changed from yellow to pink within 24 h of incubation at room temperature.

#### 2.1.4. Gold Standard Definition

A patient was defined as being *H. pylori *positive on the basis of a positive culture or, in the case of a negative culture, both a positive histological examination and a positive rapid urease test (CLO test).

#### 2.1.5. Statistical Analysis

Standard methods were used to calculate the sensitivity, specificity, positive and negative predictive values, and accuracy. All statistical analyses, data collection, and manipulation were carried out using the Statistical Package for Social Sciences (SPSS 19.0 for Windows, SPSS Inc., Chicago, IL, USA).

## 3. Results

A total of 246 patients were enrolled in this study, 106 (43.1%) were male and 140 (56.9%) were female. The age of the subjects ranged from 22 to 90 years, the mean being 53.1 ± 15.0 years. Patient characteristics, including the number infected by *H. pylori, *the number with peptic ulcers, atrophy, and intestinal metaplasia, are shown in Table 1. According to the golden standard definition, the overall *H. pylori *infection rate was 33.3%.

Table 2 shows the results of the diagnosis of *H. pylori *infection by each method in this study. Of the 246 patients, culture was positive in 75 patients (30.4%) and negative in 171 (69.6%). The CLO test (antrum and corpus) was positive in 71 patients (28.9%) and negative in 175 (71.1%). Histology (antrum and corpus) was positive in 89 patients (36.2%) and negative in 157 (63.8%).

The status of *H. pylori* infection, according to the gold standard criteria, is shown in Table 3. The sensitivity, specificity, accuracy, positive predictive value (PPV), and negative predictive value (NPV) of each diagnostic test were calculated and results are shown in Table 4. The sensitivity was good for culture (91.46%), histology (antrum), and histology (antrum and corpus) (95.12% each), but only 64.63% for the CLO test (antrum) and 69.51% for CLO test (corpus). The specificity was the best for culture, CLO test (antrum and corpus), CLO test (antrum), CLO test (corpus) (100% each), followed by histology (corpus) (96.95%), histology (antrum), andhistology (antrum and corpus) (95.12% each).

The overall accuracy was equally good in culture (97.15%), followed by the CLO test (antrum and corpus) (95.52%), histology (antrum) and histology (antrum and corpus) (95.12% each), with the poorest overall accuracy being for the CLO test (antrum) (88.21%).

## 4. Discussion

The use of a single test as the gold standard increases the error rate [16] and it is therefore recommended that the gold standard determination of infection should depend on results from two tests [17]. The gold standard in our study is a positive culture. However, if the culture is negative, positive histology and positive urease test combined correspond to acceptable criteria.

Although all the biopsies from invasive tests may give false negatives in the case of low density and patchy distribution of *H. pylori*, both histology and culture were among the best-performing tests in our study. The sensitivity of *H. pylori* culture is an important issue, and the method is not routinely recommended as the primary tool for identifying the presence of the infection [18]. However, it is becoming increasingly important in populations with high antibacterial resistance [19]. We found 91.4% sensitivity, 100% specificity, and 97.15% accuracy with this method.

Histology is a highly sensitive method for determining *H. pylori *infection and also provides insight into the status of the gastric mucosa. However, the disadvantages of this technique depend not only on the quality of the biopsy specimens, but also the expertise of the pathologists [20]. Our study demonstrated that two biopsy specimens from the antrum and one biopsy from the incisura angularis provided high diagnostic sensitivity and specificity, with an accuracy greater than 90%. But histological examination of two corpus biopsy specimens showed low sensitivity. Sampling errors, insufficient bacterial load [21], bacterial clearance [13], and patchy bacterial distribution are common causes of false negative results in corpus biopsy histology. Furthermore, biopsy specimens from both antrum and corpus showed the same sensitivity as compared with an antrum biopsy in dyspeptic patients. This is because when corpus *H. pylori* infection occurs, antrum *H. pylori* infection exists already. Our studies have identified high antral biopsy histology performance.

In the presence of *H. pylori *urease, urea is hydrolyzed, leading to a rise in pH and color change of the pH indicator (phenol red). The CLO test has been established for more than a decade and used as the main standard for rapid urease tests. The test has good performance, with a sensitivity of 89.6%, a specificity of 100%, a PPV of 100%, and a NPV of 84.1%, and has been widely acclaimed [22]. Our data showed similar results when one antrum biopsy and one corpus biopsy were combined. But a single antrum biopsy or corpus biopsy failed to meet the expectations and demonstrated low sensitivity (64.63% and 69.51%). Some previous studies have shown false negative results in up to 50% of patients over 60 years of age [23]. Mégraud had also reported low CLO test sensitivity [24]. Woo et al. reported that increasing the number of biopsies to more than one or adding extra biopsy sites may increase the sensitivity since this probably increases the *H. pylori* load and consequently the amount of urease [25]. However, this prolongs endoscopy time and adds to the discomfort of the patient.

The major limitation of this study is a lack of global evaluation tests, such as the urea breath test or the stool *H. pylori *antigen test, which have been found reliable for the detection of the presence of *H. pylori.* Sampling bias may exist within our biopsy-based diagnostic tests, including culture, histology, and the CLO test. Furthermore, the prevalence of *H. pylori *infection was low in our study cohort. It may be that our patients were mainly from an urban area (Taipei City) and might have had a lower *H. pylori *infection rate [2].

## 5. Conclusion

Antral biopsy histology and culture are the best methods for the diagnosis of *H. pylori *infection in our cohort of patients with dyspepsia. The low sensitivity of corpus biopsy histology, and of the antral or corpus biopsy CLO test, makes these seem inappropriate for the determination of the *H. pylori *status in our population.

## Figures and Tables

**Table 1:** Demographic characteristics of patients.

Age-year, mean (range) y/o	53.1 (22–90)	
Gender-number		
Male	106	43.1%
Female	140	56.9%
*H. pylori* status		
Infection	82	33.3%
Noninfection	164	66.7%
Peptic ulcer-number		
Gastric ulcer	77	31.3%
Duodenal ulcer	35	14.2%
Antrum atrophy		
Normal to mild	244	99.2%
Moderate to severe	2	0.8%
Corpus atrophy		
Normal to mild	237	96.3%
Moderate to severe	9	3.7%
Antrum intestinal metaplasia		
Normal to mild	221	89.8%
Moderate to severe	25	10.2%
Corpus intestinal metaplasia		
Normal to mild	242	98.4%
Moderate to severe	4	1.6%

**Table 2:** Results of detection of *H. pylori* by each test.

Test	Positive	Negative
Culture	75 (30.4%)	171 (69.6%)
Histology (antrum)	86 (35.0%)	160 (65.0%)
Histology (corpus)	68 (27.6%)	178 (73.4%)
Histology (antrum and corpus)	89 (36.2%)	157 (63.8%)
CLO test (antrum)	53 (21.5%)	193 (78.5%)
CLO test (corpus)	57 (23.2%)	189 (76.8%)
CLO test (antrum and corpus)	71 (28.9%)	175 (71.1%)

**Table 3:** Results of the detection of *H. pylori *by each test according to the “gold standard” definition.

Test	Result	*H. pylori* positive (*n*)	*H. pylori* negative (*n*)
Culture	Positive	75	0
	Negative	7	164
Histology (antrum)	Positive	78	8
	Negative	4	156
Histology (corpus)	Positive	63	5
	Negative	19	159
Histology (antrum and corpus)	Positive	78	8
	Negative	4	156
CLO test (antrum)	Positive	53	0
	Negative	29	164
CLO test (corpus)	Positive	57	0
	Negative	25	164
CLO test (antrum and corpus)	Positive	71	0
	Negative	11	164

**Table 4:** Results of the sensitivity, specificity, positive predictive value (PPV), negative predictive value (NPV), and accuracy among the tests.

Test	Sensitivity (%)	Specificity (%)	PPV (%)	NPV (%)	Accuracy (%)
Culture	91.46	100	100	95.91	97.15
Histology (antrum)	95.12	95.12	90.70	97.50	95.12
Histology (corpus)	76.83	96.95	92.65	89.33	90.24
Histology (antrum and corpus)	95.12	95.12	90.70	97.50	95.12
CLO test (antrum)	64.63	100	100	84.97	88.21
CLO test (corpus)	69.51	100	100	96.77	89.83
CLO test (antrum and corpus)	86.59	100	100	93.71	95.52

## References

[1] Vaira D, Miglioli M, Mulè P (1994). Prevalence of peptic ulcer in *Helicobacter pylori* positive blood donors. *Gut*.

[2] Lin JT, Wang JT, Wang TH, Wu MS, Lee TK, Chen CJ (1993). *Helicobacter pylori* infection in a randomly selected population, healthy volunteers, and patients with gastric ulcer and gastric adenocarcinoma. A seroprevalence study in Taiwan. *Scandinavian Journal of Gastroenterology*.

[3] Marshall BJ (1994). *Helicobacter pylori*. American Journal of Gastroenterology.

[4] Rauws EA, Tytgat GN (1990). Cure of duodenal ulcer associated with eradication of *Helicobacter pylori*. *The Lancet*.

[5] Asghar RJ, Parsonnet J (2001). *Helicobacter pylori* and risk for gastric adenocarcinoma. *Seminars in Gastrointestinal Disease*.

[6] Sipponen P (2002). Gastric cancer: pathogenesis, risks, and prevention. *Journal of Gastroenterology*.

[7] Megraud F (1997). How should *Helicobacter pylori* infection be diagnosed?. *Gastroenterology*.

[8] Talley NJ, Newell DG, Ormand JE (1991). Serodiagnosis of *Helicobacter pylori*: comparison of enzyme-linked immunosorbent assays. *Journal of Clinical Microbiology*.

[9] Oksanen A, Veijola L, Sipponen P, Schauman KO, Rautelin H (1998). Evaluation of Pyloriset Screen, a rapid whole-blood diagnostic test for *Helicobacter pylori* infection. *Journal of Clinical Microbiology*.

[10] (1983). Unidentified curved bacilli on gastric epithelium in active chronic gastritis. *The Lancet*.

[11] Bayerdorffer E, Oertel H, Lehn N (1989). Topographic association between active gastritis and *Campylobacter pylori* colonisation. *Journal of Clinical Pathology*.

[12] Genta RM, Graham DY (1994). Comparison of biopsy sites for the histopathologic diagnosis of *Helicobacter pylori*: a topographic study of *H. pylori* density and distribution. *Gastrointestinal Endoscopy*.

[13] Satoh K, Kimura K, Taniguchi Y (1998). Biopsy sites suitable for the diagnosis of *Helicobacter pylori* infection and the assessment of the extent of atrophic gastritis. *American Journal of Gastroenterology*.

[14] van Ijzendoorn MC, Laheij RJF, de Boer WA, Jansen JBMJ (2005). The importance of corpus biopsies for the determination of *Helicobacter pylori* infection. *Netherlands Journal of Medicine*.

[15] Tack J, Talley NJ, Camilleri M (2006). Functional gastroduodenal disorders. *Gastroenterology*.

[16] Vaira D, Vakil N (2001). Blood, urine, stool, breath, money, and *Helicobacter pylori*. *Gut*.

[17] (1997). Technical annex: tests used to assess *Helicobacter pylori* infection. Working party of the European *Helicobacter pylori* study group. *Gut*.

[18] Malfertheiner P, Megraud F, O’Morain C (2007). Current concepts in the management of *Helicobacter pylori* infection: the Maastricht III consensus report. *Gut*.

[19] Glupczynski Y (1998). Microbiological and serological diagnostic tests for *Helicobacter pylori*: an overview. *Acta Gastro*.

[20] Mégraud F (1996). Advantages and disadvantages of current diagnostic tests for the detection of *Helicobacter pylori*. *Scandinavian Journal of Gastroenterology*.

[21] Korstanje A, van Eeden S, Offerhaus GJA (2006). The 13carbon urea breath test for the diagnosis of *Helicobacter pylori* infection in subjects with atrophic gastritis: evaluation in a primary care setting. *Alimentary Pharmacology and Therapeutics*.

[22] Cutler AF, Havstad S, Ma CK, Blaser MJ, Perez-Perez GI, Schubert TT (1995). Accuracy of invasive and noninvasive tests to diagnose *Helicobacter pylori* infection. *Gastroenterology*.

[23] Abdalla AM, Sordillo EM, Hanzely Z (1998). Insensitivity of the CLOtest for *H. pylori*, especially in the elderly. *Gastroenterology*.

[24] Mégraud F (1997). A growing demand for *Helicobacter pylori* culture in the near future?. *Italian Journal of Gastroenterology and Hepatology*.

[25] Woo JS, El-Zimaity HMT, Genta RM, Yousfi MM, Graham DY (1996). The best gastric site for obtaining a positive rapid urease test. *Helicobacter*.

